# The Diverse Clinical Spectrum of Noonan Syndrome in Pediatric Endocrinology: From Classical to Atypical Phenotypes

**DOI:** 10.3390/jcm15197561

**Published:** 2026-09-29

**Authors:** Sara Aszkiełowicz, Anna Łupińska, Arkadiusz Zygmunt, Renata Stawerska

**Affiliations:** 1 Department of Endocrinology and Metabolic Diseases, Polish Mother’s Memorial Hospital—Research Institute of Lodz, 93-338 Lodz, Poland; sara.aszkielowicz@iczmp.edu.pl (S.A.); arkadiusz.zygmunt@umed.lodz.pl (A.Z.); renata.stawerska@umed.lodz.pl (R.S.); 2 Department of Developmental Age and Adult Endocrinology, Medical University of Lodz, 93-338 Lodz, Poland

**Keywords:** noonan syndrome, phenotypic variability, short stature, growth impairment, pediatric endocrinology, atypical phenotype

## Abstract

**Background/Objectives**: Noonan syndrome (NS) is characterized by marked phenotypic variability. Although the classical phenotype includes short stature, craniofacial dysmorphism, skeletal abnormalities, and congenital heart defects, some patients present with subtle or atypical manifestations, making clinical recognition challenging. **Methods**: This retrospective case series included six pediatric patients with molecularly confirmed NS managed at the Department of Endocrinology and Metabolic Diseases, Polish Mother’s Memorial Hospital-Research Institute, Lodz, Poland. Medical records were reviewed for auxological data, phenotypic features, endocrine and skeletal assessment, cardiovascular findings, associated conditions, and multidisciplinary management. The aim of this study was to characterize the phenotypic diversity of NS in pediatric endocrine practice, with particular emphasis on growth patterns, endocrine and skeletal manifestations, and atypical clinical presentations. **Results**: The six patients demonstrated markedly heterogeneous clinical presentations. One boy exhibited the classical phenotype with severe short stature and pulmonary valve stenosis, whereas a girl with typical NS features maintained relatively preserved growth around the 3rd percentile. Another patient presented with progressive resorption of permanent incisor roots and a coexisting pathogenic NBN variant. A boy with LZTR1-associated NS and Klinefelter syndrome (47,XXY) demonstrated a blended phenotype and height between the 25th and 50th percentiles. Two girls lacked characteristic NS dysmorphism and presented predominantly with growth impairment, accompanied by markedly low bone mass in one case and a prolonged diagnostic pathway in the other. GH deficiency was not identified in the evaluated patients, and rhGH therapy was initiated in three patients. **Conclusions**: NS encompasses a broad clinical spectrum, ranging from classical phenotypes to atypical presentations dominated by growth, skeletal, or dental abnormalities. Neither the absence of characteristic dysmorphism nor relatively preserved growth excludes the diagnosis, while coexisting genetic conditions may further modify the phenotype. Awareness of this heterogeneity is essential for appropriate recognition and individualized multidisciplinary care. In selected children with otherwise unexplained growth or atypical clinical findings, consideration of NS and genetic evaluation may help establish a unifying diagnosis and potentially shorten the diagnostic pathway.

## 1. Introduction

Noonan Syndrome (NS) is a multisystem developmental disorder belonging to the group of diseases collectively referred to as RASopathies, caused by dysregulation of the RAS/mitogen-activated protein kinase (RAS/MAPK) signaling pathway. The estimated prevalence ranges from 1:1000 to 1:2500 live births, making NS one of the most common syndromic causes of congenital heart disease and short stature [1,2]. Since the original description by Jacqueline Noonan in the 1960s [3], the phenotypic spectrum has broadened substantially, and the disorder is now recognized as clinically heterogeneous. More broadly, endocrine and metabolic disorders may present with heterogeneous, multisystem phenotypes, emphasizing the importance of comprehensive assessment extending beyond abnormalities of a single organ or hormonal axis [4]. This approach is particularly relevant to syndromic conditions such as NS, in which endocrine manifestations coexist with cardiovascular, skeletal, developmental, and other systemic features.

The molecular background of NS involves pathogenic variants in genes encoding proteins that regulate intracellular signal transduction through the RAS/MAPK cascade. The most frequently affected gene is PTPN11, accounting for approximately 40–50% of cases, followed by SOS1, RAF1, RIT1, KRAS, and LZTR1 [2,5,6]. More recently, studies investigating unresolved RASopathy phenotypes identified both autosomal dominant and autosomal recessive forms of LZTR1-associated NS, highlighting the complexity of genotype–phenotype relationships and the role of altered RAS protein ubiquitination in disease pathogenesis [7,8,9].

Clinically, NS is characterized by highly variable expressivity. Typical manifestations include short stature, congenital heart defects, craniofacial dysmorphism, broad or webbed neck, chest wall deformities, cryptorchidism, lymphatic abnormalities, bleeding diathesis, and neurodevelopmental impairment [2]. Cardiovascular involvement is observed in approximately 50–80% of patients, with pulmonary valve stenosis and hypertrophic cardiomyopathy being the most common abnormalities. Growth impairment represents another major clinical concern and may result from a complex interplay between altered GH secretion, partial GH resistance, impaired IGF-1 signaling, nutritional factors, and delayed puberty [1,6,10,11].

Although classical phenotypic features facilitate diagnosis in many children, atypical presentations are increasingly recognized owing to wider access to molecular testing. Some individuals present primarily with isolated short stature or subtle dysmorphic features, whereas others exhibit overlapping phenotypes caused by coexisting pathogenic variants in unrelated genes. The expanding phenotypic spectrum of NS poses new diagnostic challenges for pediatric endocrinologists, who are increasingly confronted with patients lacking classical syndromic features. The present case series illustrates this clinical heterogeneity by describing six children with genetically confirmed NS who presented with markedly different phenotypes, including isolated short stature, severe root resorption of permanent teeth, coexistence of Klinefelter syndrome, and classical NS with congenital heart disease. Taken together, these cases highlight the broad phenotypic spectrum of NS and underscore the diagnostic challenges posed by atypical presentations, particularly in children referred for evaluation of growth abnormalities and other endocrine manifestations.

## 2. Materials and Methods

We conducted a retrospective case series of six pediatric patients with molecularly confirmed NS, selected to illustrate the broad phenotypic spectrum encountered in pediatric endocrinology, who were managed at the Department of Endocrinology and Metabolic Diseases, Polish Mother’s Memorial Hospital–Research Institute in Lodz, Poland. Patient data were de-identified to ensure confidentiality. Written informed consent was obtained from the parents or legal guardians. The study was conducted in accordance with the Declaration of Helsinki and was approved by the Bioethics Committee of the Polish Mother’s Memorial Hospital–Research Institute (approval no. 32/2026; 24 March 2026). Written informed consent for the publication of anonymized clinical photographs was additionally obtained from the parents or legal guardians of the patients whose photographs are presented in this manuscript.

Clinical data were retrospectively reviewed and included detailed medical history, physical examination, auxological assessment, and documentation of dysmorphic features, musculoskeletal abnormalities, cardiovascular findings, and relevant comorbidities. Height and weight were measured according to standard clinical procedures, and BMI was calculated as weight in kilograms divided by the square of height in meters. Height measurements were plotted on sex- and age-specific height percentile charts developed by Palczewska and Niedźwiecka based on body height measurements obtained in a cross-sectional anthropometric study of Polish children and adolescents and routinely used for growth assessment in Polish pediatric practice [12]. Height was additionally expressed as a standard deviation score (hSDS), where applicable. Mid-parental height (MPH) was calculated from parental heights and indicated on individual growth charts when parental height data were available.

Laboratory investigations were performed according to individual clinical indications and included assessment of thyroid function, the GH–IGF-1 axis, adrenal and gonadal function, calcium–phosphate metabolism, screening for celiac disease, and routine hematological and biochemical parameters. Additional endocrine investigations, including pharmacological GH stimulation tests and other dynamic endocrine tests, were performed when clinically indicated. Relevant imaging studies and multidisciplinary specialist consultations were reviewed according to the individual clinical presentation.

DXA was performed in selected patients when clinically indicated. DXA measurements were performed using the Hologic Horizon Bone Densitometry System (Hologic Inc., Marlborough, MA, USA). Areal bone mineral density (aBMD) was assessed at two skeletal sites: total body less head (TBLH) and lumbar spine (LS). Results were expressed as age- and sex-specific aBMD Z-scores. In patients with short stature (hSDS ≤ −2.0), aBMD Z-scores were additionally adjusted for height to account for the potential influence of smaller body and bone size on DXA-derived measurements. Height-adjusted Z-scores (HAZ) were calculated according to the previously described method [13], using the following equation:

BMD_HAZ_ = BMD_for age_ Z-score − HAZ-predicted BMD Z-score

Both age-specific aBMD Z-scores and height-adjusted values, where applicable, were considered in the assessment of bone status.

The diagnosis of NS was molecularly confirmed in all patients as part of routine clinical diagnostic evaluation. Genetic findings relevant to the observed clinical phenotypes are presented descriptively. Molecular genetic testing was performed as part of routine clinical diagnostic evaluation, and the variant classifications reported in this study reflect the interpretations provided by the performing diagnostic laboratories. As the complete laboratory-level evidence underlying the ACMG/AMP classifications was not available to the authors, the reported variants were not independently reclassified. Detailed molecular methodology was therefore beyond the scope of the present case series. Treatment decisions, including the initiation of recombinant growth hormone (rhGH) therapy, were based on individual clinical indications and established pediatric endocrinology practice. Patients were followed longitudinally, with regular assessment of growth, laboratory parameters, and organ-specific surveillance tailored to their clinical phenotype and associated comorbidities.

## 3. Results

A summary of the major clinical findings, genetic results, and therapeutic interventions is presented in Table 1.

### 3.1. Patient 1: Classical Noonan Syndrome Phenotype

An 8-year-old boy with molecularly confirmed NS caused by a pathogenic PTPN11 variant was repeatedly admitted to the Department because of severe short stature (−4.49 hSDS) requiring comprehensive endocrine assessment and long-term follow-up. He also remained under regular cardiological care because of pulmonary valve stenosis. His mother’s height was 152 cm and his father’s height was 177 cm.

Physical examination demonstrated the classical phenotypic features of NS, including prominent frontal bossing, hypertelorism, ptosis, a depressed nasal bridge, low-set ears, a webbed neck, pectus excavatum, and generalized muscular hypotonia, representing the most recognizable clinical phenotype within our cohort.

During successive endocrine evaluations, comprehensive investigations repeatedly excluded endocrine causes of growth impairment. Growth hormone stimulation testing consistently demonstrated normal GH secretion, thereby excluding somatotropic pituitary insufficiency (Table 2). Brain and pituitary MRI revealed no structural abnormalities. Despite preserved pituitary function, the patient continued to exhibit profound growth failure.

Given the severity of short stature and the absence of contraindications identified during cardiological assessment, rhGH therapy was initiated. The patient remains under regular multidisciplinary follow-up by pediatric endocrinology, cardiology, and clinical genetics.

### 3.2. Patient 2: Classical Phenotype with Relatively Preserved Linear Growth

A 4-year-old girl with molecularly confirmed NS was admitted to the Department for evaluation of her growth pattern. In contrast to most patients in the present series, the diagnosis of NS had been established early, during the first year of life, following identification of a heterozygous pathogenic variant in the PTPN11 gene. The patient exhibited a characteristic NS phenotype, including hypertelorism, downslanting palpebral fissures, low-set and posteriorly rotated ears, a broad/depressed nasal bridge, a short webbed neck, and a broad chest with widely spaced nipples. She remained under multidisciplinary follow-up by pediatric cardiology because of Wolff–Parkinson–White syndrome, as well as by pediatric neurology, endocrinology, and physiotherapy. Despite the characteristic syndromic phenotype, the patient exhibited only mild short stature, with preserved growth velocity and height consistently tracking around the 3rd percentile, although markedly below her genetic height potential. Of note, both parents were relatively tall, with maternal and paternal heights of 174 cm and 180 cm, respectively. Her growth trajectory remained harmonious, without evidence of progressive growth deceleration. Comprehensive endocrine assessment did not identify a hormonal cause of growth impairment. Pharmacological GH stimulation testing demonstrated normal GH secretion, serum IGF-1 concentrations were within the age- and sex-specific reference range, and thyroid and adrenal function were normal (Table 2). Bone age was approximately concordant with chronological age. The patient remains under regular multidisciplinary surveillance, showing a stable growth trajectory.

### 3.3. Patient 3: Atypical Dental Presentation Revealing Noonan Syndrome

A 14-year-old boy was referred because of progressive external root resorption of the permanent incisors, initially identified during routine dental evaluation. This unusual dental finding represented the first clinical manifestation prompting further diagnostic investigations. The patient had normal psychomotor development and did not exhibit characteristic craniofacial dysmorphism, congenital heart disease, or other clinical features suggestive of NS. His height remained between the 10th and 25th percentiles throughout follow-up.

Particular attention was given to calcium–phosphate metabolism and bone status because of the progressive dental abnormalities. Serum total calcium [2.55 mmol/L; reference range (RR), 2.20–2.70], ionized calcium [1.30 mmol/L; RR, 1.20–1.32], and phosphate [1.74 mmol/L; RR, 1.09–2.00] concentrations were within the reference ranges, as were serum creatinine, PTH [27.40 pg/mL; RR, 15–65], and alkaline phosphatase activity [311 U/L; RR, 130–534]. Vitamin D status was adequate, and supplementation with 2000 IU/day was continued. Twenty-four-hour urine collection demonstrated normocalciuria and low urinary phosphate excretion, with fractional calcium excretion (FeCa) of 0.58–0.80%, tubular reabsorption of phosphate (TRP) of 97%, and TmP/GFR of 2.23–2.51. Overall, these findings provided no evidence of renal calcium or phosphate wasting. DXA demonstrated bone mass within the expected range for age, with an aBMD_for-age_ Z-score of −0.5 at the TBLH and −0.8 at the lumbar spine. Selected biochemical and densitometric parameters of bone and mineral metabolism are presented in Table 2. Pituitary MRI showed no structural abnormalities.

Despite extensive laboratory, metabolic, endocrine and imaging investigations, no unifying diagnosis explaining the progressive dental lesions could be established. Therefore, the patient was referred for genetic evaluation. During his initial endocrine assessment, molecular testing had already been initiated, and at a subsequent follow-up visit he returned with the results. NGS identified the heterozygous PTPN11 variant NM_002834.5:c.794G>A (p.Arg265Gln), classified as pathogenic according to ACMG/AMP criteria in the original diagnostic laboratory report, thereby confirming the molecular diagnosis of NS. The PTPN11 variant was subsequently confirmed by Sanger sequencing, and segregation analysis indicated a de novo occurrence. A heterozygous pathogenic variant in the NBN gene was also identified; however, this finding did not establish a diagnosis of Nijmegen breakage syndrome. Cardiological assessment, including electrocardiography and echocardiography, revealed no abnormalities. The patient remains under regular multidisciplinary follow-up by pediatric endocrinology, dentistry, clinical genetics, and pediatric oncology.

### 3.4. Patient 4: Blended Phenotype Resulting from Dual Genetic Diagnoses

An 8-year-old boy with Klinefelter syndrome (47,XXY) and molecularly confirmed LZTR1-associated Noonan syndrome was referred to our Department for comprehensive endocrine evaluation and further follow-up. The LZTR1 variant had been classified as pathogenic in the original diagnostic genetic report. Klinefelter syndrome had been diagnosed in 2023, whereas the molecular diagnosis of NS was established subsequently, in early 2024.

Prenatal ultrasonography had demonstrated increased nuchal translucency and bilateral pelvicalyceal dilatation. During early childhood, the patient experienced recurrent upper respiratory tract infections and remained under pediatric urological follow-up because of bilateral retractile testes. Although dysmorphic features compatible with NS had been present since early childhood, the diagnosis of NS was established only after the diagnosis of Klinefelter syndrome.

Physical examination revealed ptosis, hypertelorism, low-set ears, generalized hypotonia, and mild developmental delay. He was prepubertal (Tanner stage I). Notably, his height consistently remained between the 25th and 50th percentiles throughout childhood, despite the coexistence of two genetic conditions with potentially distinct effects on linear growth.

Comprehensive endocrine evaluation revealed normal thyroid function and serum IGF-1 levels. Assessment of the hypothalamic–pituitary–adrenal axis showed a morning ACTH level of 26.9 pg/mL and a preserved circadian cortisol rhythm, with serum cortisol values of 14.9 µg/dL at 08:00 and 1.0 µg/dL at midnight. Morning serum prolactin was 5.5 ng/mL. The patient was prepubertal, with gonadotropin and androgen levels appropriate for his developmental stage: FSH 0.6 IU/L, LH <0.1 IU/L, and testosterone < 0.025 ng/mL. Assessment of carbohydrate metabolism showed no evidence of glucose intolerance, while parameters of calcium–phosphate metabolism remained within the reference ranges.

**Figure 1:**
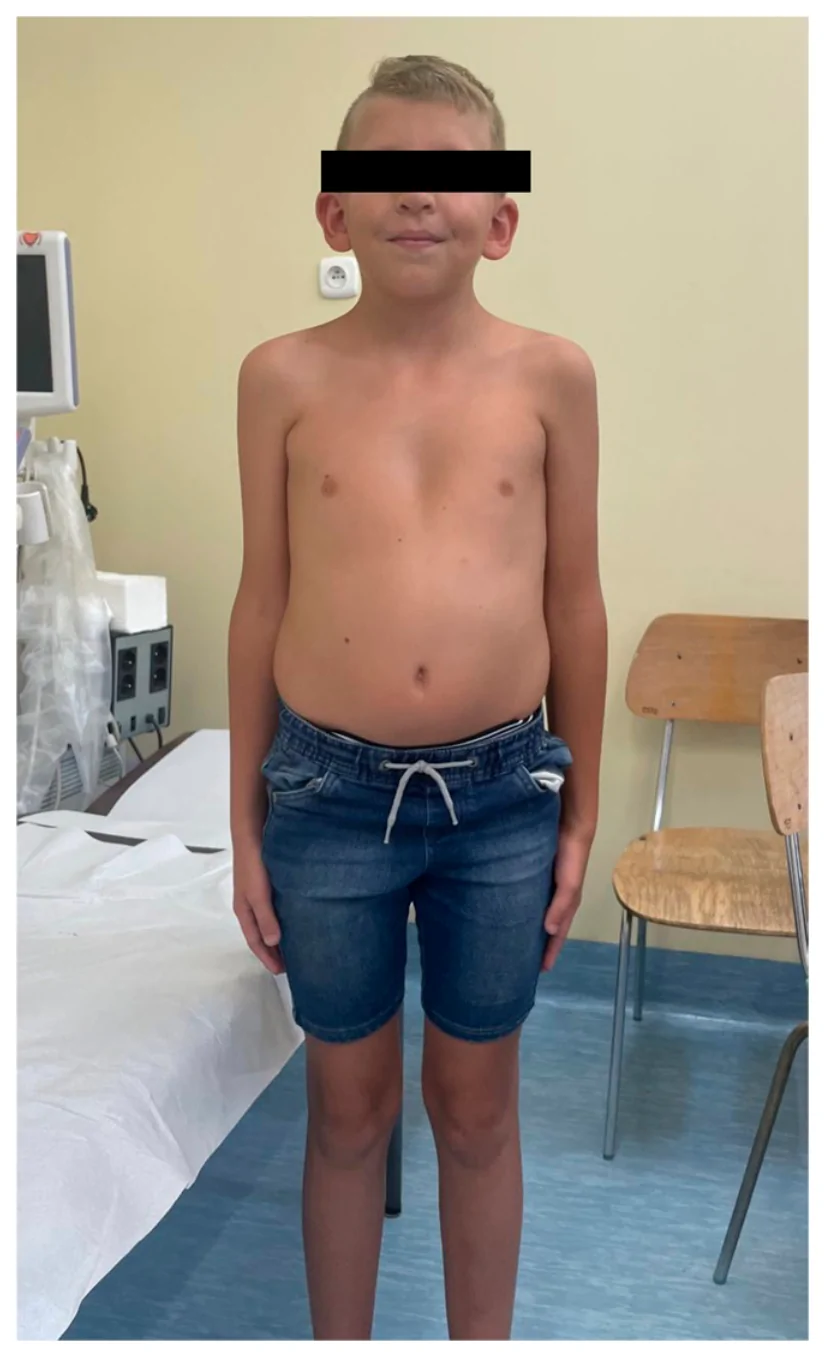
Clinical appearance of Patient 4 with molecularly confirmed Noonan syndrome and a 47,XXY karyotype.

The patient remained under regular cardiological follow-up because of congenital mitral valve insufficiency, with no additional cardiovascular abnormalities requiring intervention. He also continued multidisciplinary follow-up by pediatric endocrinology, clinical genetics, and pediatric urology (Figure 1).

### 3.5. Patient 5: Oligosymptomatic Noonan Syndrome with Severe Short Stature and Low Bone Mass

A 14-year-old girl was referred because of severe short stature. Her height was below the 3rd percentile (−2.9 hSDS), with a BMI SDS of −3.27. Assessment of bone status confirmed markedly low bone mass, with an aBMD_for-age_ Z-score of −4.80 at the TBLH and −4.90 at the lumbar spine (Table 2). Although aBMD was markedly reduced, the patient had no history of fractures. Biochemical assessment of bone turnover showed a serum osteocalcin concentration of 89.4 ng/mL and β-CrossLaps (β-CTX) concentration of 1481.0 pg/mL. At presentation, she did not exhibit characteristic facial dysmorphism or previously recognized congenital heart disease.

Because of persistent unexplained growth failure accompanied by low bone mass, the patient underwent a prolonged, stepwise diagnostic evaluation requiring several hospital admissions. During her first endocrine admission in September 2021, comprehensive investigations excluded endocrine causes of growth impairment, including growth hormone deficiency. At that time, she was prepubertal (Tanner stage I), and her serum IGF-1 concentration was reduced at 95.3 ng/mL (RR, 140–468 ng/mL). Conventional cytogenetic analysis demonstrated a normal female karyotype (46,XX).

As growth failure persisted, the patient was readmitted in April 2023. Repeat GH stimulation testing again demonstrated normal GH secretion, with a maximum stimulated GH concentration of 12.78 ng/mL, further excluding growth hormone deficiency. Despite preserved GH secretion, serum IGF-1 remained below the reference range at 134.0 ng/mL (RR, 151–485 ng/mL). During a subsequent endocrine admission in July 2023, serum IGF-1 had increased to 172.3 ng/mL (RR, 151–485 ng/mL). Given the previously persistent reduction in IGF-1 concentrations despite normal stimulated GH secretion, an IGF-1 generation test was performed. IGF-1 increased from 172.3 ng/mL at baseline to 297.9 ng/mL (RR, 151–485 ng/mL) after 5 days of recombinant GH administration at a dose of 0.1 IU/kg/day, demonstrating an appropriate IGF-1 response and providing no evidence of severe primary IGF-1 deficiency. Additional endocrine investigations performed throughout the diagnostic process excluded hypercortisolism and hypogonadism as potential contributors to growth impairment and low bone mass.

As part of the multidisciplinary work-up, the patient had also been admitted twice to the Department of Pediatric Gastroenterology, in October 2019 and April 2021. Gastroesophageal reflux disease was diagnosed, and genetic testing demonstrated a predisposition to celiac disease; however, no gastrointestinal disorder capable of explaining the severity of her growth failure or low bone mass was identified. She also remained under regular neurological follow-up because of epilepsy and was referred to a specialized pediatric osteoporosis clinic owing to markedly low bone mass.

**Figure 2:**
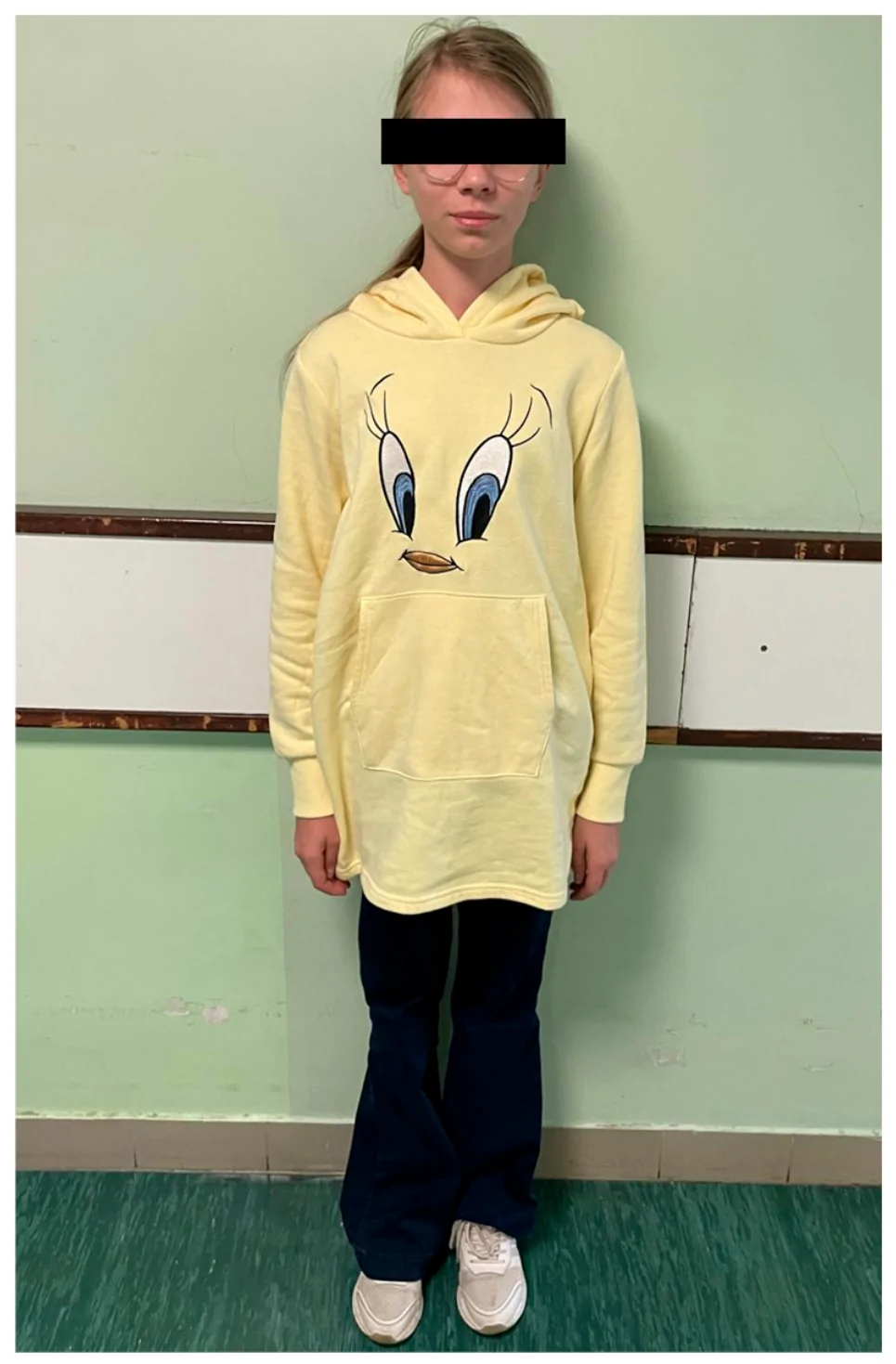
Clinical appearance of Patient 5, in whom characteristic craniofacial features of Noonan syndrome were not evident on clinical examination.

Despite repeated evaluations involving several pediatric specialties and multiple hospital admissions over an extended period, no unifying diagnosis explaining the combination of severe short stature and low bone mass could initially be established. Further genetic evaluation identified a pathogenic PTPN11 variant, confirming the diagnosis of NS. Following the diagnosis, targeted cardiological assessment revealed a thick false tendon of the left ventricle and a Chiari network within the right atrium, neither of which had been recognized previously. Because of progressive growth failure, rhGH therapy was subsequently initiated (Figure 2).

### 3.6. Patient 6: Oligosymptomatic Noonan Syndrome with Short Stature as the Predominant Clinical Feature

A girl was referred to the Department of Endocrinology and Metabolic Diseases because of short stature accompanied by progressive growth deceleration, first noted at approximately 7 years of age. She had no congenital heart defects, developmental delay, or characteristic craniofacial features suggestive of NS. Family history was notable for short stature in her mother, whose adult height was 160 cm (−0.97 hSDS), while the father’s height was 185 cm. The mother had been treated during childhood with rhGH and triptorelin because of rapidly progressive central precocious puberty associated with an unfavorable height prognosis.

Because of persistent unexplained growth failure, the patient underwent a stepwise endocrine evaluation requiring four hospital admissions in February, June, and November 2023 and April 2024. At the initial assessment, she was at Tanner stage II, progressing to Tanner stage II/III by April 2024. Serum IGF-1 concentrations remained consistently below the age-specific reference ranges throughout this period: 107.2 ng/mL (RR, 132–451) in February 2023, 89.8 ng/mL (RR, 132–451) in June 2023, 114.6 ng/mL (RR, 140–468) in November 2023, and 129.6 ng/mL (RR, 140–468) in April 2024. Pharmacological GH stimulation tests with clonidine and glucagon, performed on two separate occasions, demonstrated normal GH secretion, with a maximum stimulated GH concentration of 16.94 ng/mL, thereby excluding GH deficiency. Given the persistent reduction in IGF-1 concentrations despite preserved stimulated GH secretion, an IGF-1 generation test was performed. IGF-1 increased from 135.1 ng/mL (RR, 140–468 ng/mL) at baseline to 185.1 ng/mL (RR, 140–468 ng/mL) after 5 days of recombinant GH administration at a dose of 0.1 IU/kg/day, demonstrating an appropriate IGF-1 response and providing no evidence of severe primary IGF-1 deficiency.

Serial pituitary MRI examinations demonstrated persistently reduced contrast enhancement of the anterior pituitary without evidence of a structural lesion. Conventional cytogenetic analysis revealed a normal female karyotype (46,XX). Because of the coexistence of persistently low IGF-1 concentrations and low body weight (BMI SDS −1.10), the patient was also evaluated in the Department of Pediatric Gastroenterology. Laboratory findings raised suspicion of chronic gastritis; however, no gastrointestinal disorder capable of explaining the severity of her growth impairment was identified. Thus, despite repeated endocrine and multidisciplinary evaluations, the etiology of her short stature remained unexplained.

Given the persistent short stature and progressive growth deceleration, together with repeatedly reduced IGF-1 concentrations despite preserved stimulated GH secretion, further genetic evaluation was undertaken. NGS identified a heterozygous pathogenic variant in the PTPN11 gene, confirming the diagnosis of NS. Notably, despite the molecular diagnosis, the patient did not exhibit the characteristic phenotypic features traditionally associated with NS. Cascade genetic testing subsequently identified the same pathogenic PTPN11 variant in her brother, who likewise lacked classical NS features and presented with short stature (−2.00 hSDS), illustrating substantial intrafamilial phenotypic variability. The patient’s mother is currently undergoing molecular genetic evaluation.

**Figure 3:**
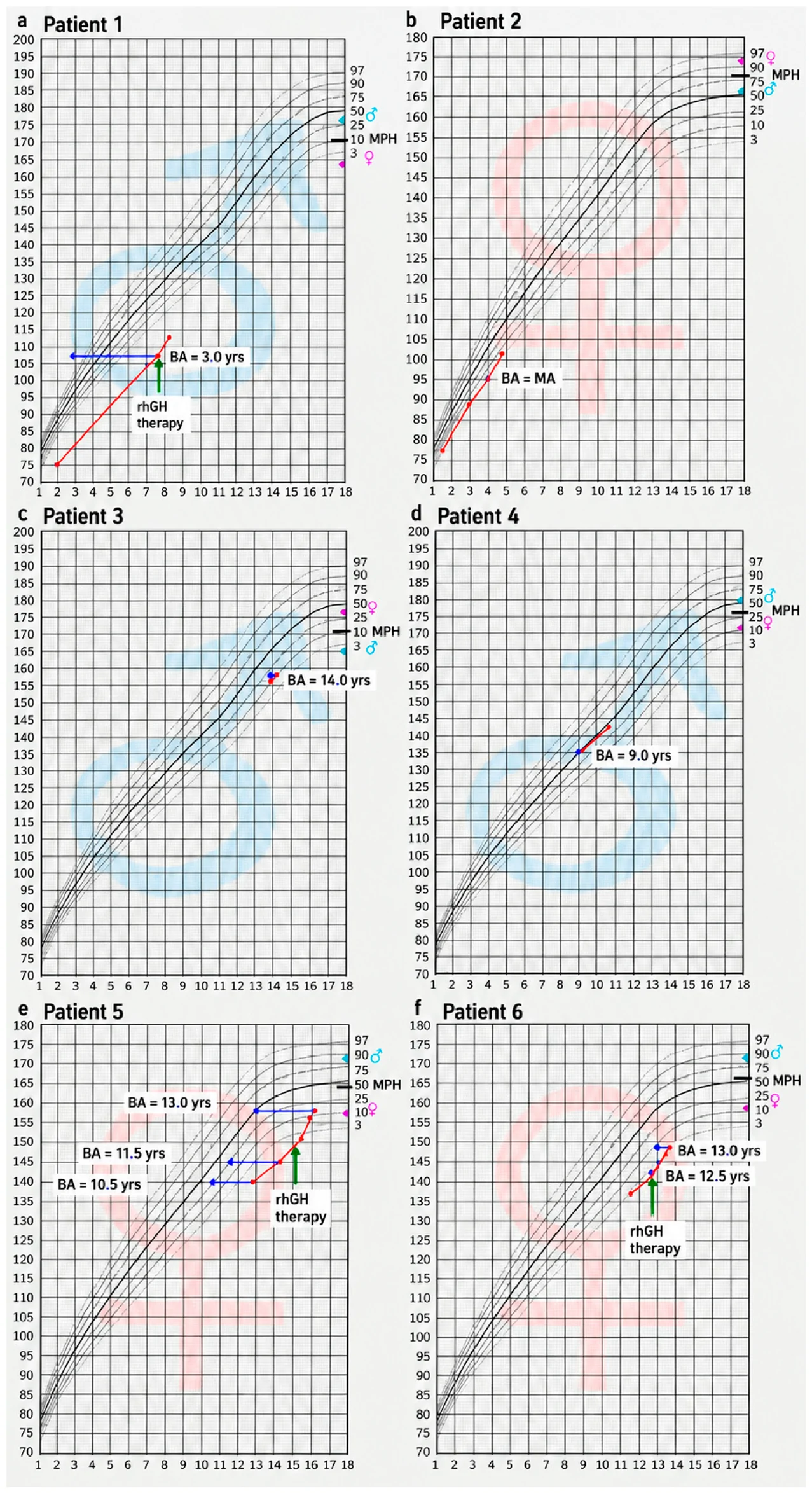
Growth charts of Patients 1–6 (**a**–**f**) included in the case series, based on the sex- and age-specific Polish reference charts developed by Palczewska and Niedźwiecka. Mid-parental height (MPH), available bone-age (BA) assessments, and the initiation of recombinant human growth hormone (rhGH) treatment are indicated where applicable.

Growth charts of all six patients are presented in Figure 3, highlighting the marked auxological variability within the cohort, ranging from severe short stature to height within the normal range.

## 4. Discussion

The present case series highlights the substantial clinical heterogeneity of NS, encompassing a spectrum from classical syndromic presentations to atypical and oligosymptomatic phenotypes in which growth disturbances, skeletal abnormalities, or other less common manifestations predominate. Although NS is traditionally associated with characteristic craniofacial features, congenital heart defects, and short stature, the patients described in our series exhibited considerable variability in both the type and severity of clinical manifestations. This diversity underscores the diagnostic challenges posed by NS, particularly when its hallmark features are absent, subtle, or overshadowed by less typical findings [8].

Growth impairment remains one of the most clinically significant manifestations of NS. Previous studies demonstrated that mean adult height in untreated individuals with NS is approximately 1.5–2 standard deviations below the population mean, although severity varies substantially depending on genotype [1,6,10]. Variants in PTPN11 are particularly associated with more pronounced postnatal growth retardation and lower circulating IGF-1 concentrations, whereas patients carrying SOS1 variants often exhibit milder growth disturbances and relatively preserved stature [5,6]. These genotype-dependent differences likely reflect distinct effects of individual pathogenic variants on intracellular signaling through the RAS/MAPK pathway.

The pathophysiology of growth failure in NS is complex and multifactorial. Proposed mechanisms include altered GH secretion, neurosecretory dysfunction, delayed puberty, nutritional impairment, chronic cardiovascular disease, partial GH resistance, and disturbed IGF-1 signaling [10,11]. Binder et al. demonstrated that individuals with PTPN11-associated NS exhibit reduced sensitivity to endogenous GH, supporting the concept of partial GH resistance mediated through excessive activation of the RAS/MAPK cascade [11]. Hyperactivation of this pathway may impair post-receptor GH signaling and suppress hepatic IGF-1 synthesis, thereby contributing to impaired longitudinal growth despite apparently normal endocrine investigations.

This mechanism appears particularly relevant to the growth patterns observed in our cohort. In patients with marked short stature, repeated pharmacological stimulation tests demonstrated preserved GH secretion, whereas reduced IGF-1 concentrations were documented in Patients 5 and 6. This discordance between stimulated GH secretion and circulating IGF-1 levels may be consistent with impaired peripheral responsiveness to GH rather than primary pituitary dysfunction and may contribute to growth impairment in NS. Nevertheless, growth failure in NS is complex and multifactorial, and altered GH sensitivity represents only one of several potential contributing mechanisms. Nutritional status, congenital heart disease, delayed pubertal development, and other syndrome-related factors may additionally influence linear growth. Accordingly, preserved GH secretion does not necessarily exclude functional alterations within the GH–IGF-1 axis. The combination of normal stimulated GH secretion and reduced IGF-1 concentrations observed in some of our patients may therefore provide a plausible pathophysiological context for impaired growth despite otherwise unremarkable endocrine findings. Recognition of this pattern may be particularly relevant in patients with atypical or oligosymptomatic NS, in whom short stature or progressive growth deceleration may constitute the predominant clinical manifestation.

Patient 5 and Patient 6 illustrate the diagnostic challenges associated with atypical or oligosymptomatic NS. Severe short stature represented the predominant manifestation in the absence of characteristic dysmorphism or congenital heart disease. Similar atypical presentations have been described in previous reports [14,15], emphasizing that NS may remain clinically unrecognized when classical facial features are absent or subtle. Romano et al. highlighted that isolated short stature may occasionally constitute the principal manifestation during childhood, especially in patients with milder molecular subtypes or before development of the full syndromic phenotype [2]. These observations reinforce the importance of considering RASopathies in the differential diagnosis of unexplained idiopathic short stature, particularly when auxological impairment is disproportionate or associated with subtle skeletal or developmental abnormalities.

The reduced bone mineral density observed in our patient is in keeping with previous reports suggesting that impaired bone mineralization may be an underrecognized skeletal manifestation of NS. Choudhry et al. identified low bone mass in 50% of children with NS evaluated by DXA and reported a significantly reduced mean total-body BMD Z-score, indicating that abnormalities in bone mineralization may form part of the NS phenotype [16]. Given the important role of the RAS/MAPK pathway in bone homeostasis, dysregulation of this pathway has been proposed as a potential mechanism underlying altered skeletal mineralization in NS [16]. Nevertheless, the available evidence remains limited, and other factors, including short stature, delayed puberty, nutritional status, reduced physical activity, and hormonal disturbances, may also contribute to impaired bone mineralization. In Patient 5, the markedly reduced BMI represents an important additional factor that may have contributed to the observed reduction in bone mass.

In this context, the markedly reduced BMD observed in our patient may reflect a broader skeletal component of the NS phenotype rather than an isolated abnormality. Assessment of bone health may therefore be considered in selected patients with NS, particularly in those with significant growth impairment or additional risk factors for skeletal complications. However, further studies are needed to establish the prevalence and clinical significance of reduced bone mass in this population and to determine the optimal approach to its monitoring.

From a practical clinical perspective, the diagnostic evaluation of children with oligosymptomatic NS may be particularly challenging when short stature is the predominant presenting feature. In the absence of typical dysmorphic features or congenital heart disease, these patients may initially be diagnosed with idiopathic short stature and undergo an extended endocrine work-up before the underlying condition is recognized. The present case highlights that severe growth impairment can be the main clinical manifestation of NS, even in the presence of less typical findings, such as reduced bone mineral density. Consequently, NS should be considered in the differential diagnosis of children presenting with persistent, unexplained, proportionate short stature, especially when growth impairment is associated with skeletal abnormalities or other subtle multisystem manifestations. Recognition of these atypical phenotypes may contribute to an earlier diagnosis and enable appropriate multidisciplinary follow-up.

Patient 4 carried both a pathogenic LZTR1 variant and a 47,XXY karyotype consistent with Klinefelter syndrome. The coexistence of these disorders may have contributed to the relatively preserved growth pattern observed in this patient, contrasting with the short stature typically associated with NS. Klinefelter syndrome is generally associated with tall stature resulting from SHOX overdosage [17]. Therefore, the simultaneous presence of both conditions may partially counterbalance their opposing effects on growth. Such blended phenotypes are increasingly recognized in the genomic era and illustrate the complexity of genotype–phenotype interpretation in modern clinical genetics. Although multilocus genomic variation has been described in various developmental disorders, reports of concomitant NS and Klinefelter syndrome remain exceptionally rare. To our knowledge, only isolated case reports have documented this combination [18], highlighting its rarity and the limited understanding of its clinical consequences. Consequently, each additional well-characterized patient contributes valuable information regarding the interaction between these two conditions and their impact on growth, pubertal development and long-term management.

Recent years have significantly expanded the understanding of LZTR1-associated NS. Johnston et al. first confirmed the existence of autosomal recessive NS associated with biallelic LZTR1 variants, thereby broadening the molecular spectrum of RASopathies [19]. Subsequently, Pagnamenta et al. further delineated dominant and recessive forms of LZTR1-associated NS and demonstrated considerable phenotypic heterogeneity among affected individuals [8]. Importantly, one of their patients additionally harbored pathogenic variants in another disease-associated gene, illustrating how multilocus pathogenic variation may generate composite phenotypes resembling those observed in our cohort.

The coexistence of a pathogenic PTPN11 variant and a heterozygous pathogenic NBN variant in Patient 3 represents one of the most clinically intriguing molecular findings in our case series. Importantly, the heterozygous NBN variant did not establish Nijmegen breakage syndrome and should be regarded as an additional molecular finding rather than a confirmed explanation for the dental phenotype. Accordingly, the progressive external root resorption should be interpreted primarily in the context of the patient’s PTPN11-associated Noonan syndrome, while any potential modifying effect of the additional NBN variant remains speculative. Notably, progressive external root resorption of the permanent incisors constituted the initial and only manifestation leading to further diagnostic evaluation and ultimately to the diagnosis of NS.

Oral and dental abnormalities are increasingly recognized as part of the phenotypic spectrum of NS and other RASopathies. In NS, reported findings include a high-arched palate, malocclusion, delayed or disturbed tooth eruption, impacted or supernumerary teeth, and other orthodontic abnormalities [20]. Similar but partly distinct dental phenotypes have been reported across other RASopathies, including abnormalities of tooth development and eruption and osteolytic or giant cell lesions, supporting a broader involvement of dysregulated RAS/MAPK signaling in craniofacial and dental homeostasis [21].

Central giant cell lesions of the jaws have also been repeatedly described in NS and related RASopathies, suggesting that altered RAS/MAPK signaling may affect local remodeling within craniofacial tissues [22,23]. Progressive external root resorption, however, has not been recognized as a characteristic manifestation of NS. To our knowledge, only a single case of cervical root resorption has been reported in a patient with genetically confirmed NS; importantly, that lesion developed following prolonged orthodontic treatment and was considered treatment-related rather than syndrome-related [24].

From a local pathophysiological perspective, root resorption represents a process of dental hard-tissue loss mediated by odontoclastic activity. Its initiation requires disruption of the protective environment of the root surface, allowing recruitment and activation of resorptive cells within the local periodontal microenvironment [25].

Cellular and molecular studies of external root resorption further indicate that the balance between local pro-resorptive and reparative processes involves interactions among clastic cells, macrophages, inflammatory mediators, cementum, and other cells of the dental microenvironment [26]. In this context, the spectrum of dental and jaw abnormalities reported in RASopathies raises the hypothesis that dysregulated RAS/MAPK signaling could modify local tissue homeostasis and thereby influence susceptibility to root resorption [21,27].

However, a direct mechanistic link between PTPN11-associated RAS/MAPK dysregulation and odontoclast-mediated external root resorption has not been demonstrated, and this proposed mechanism therefore remains speculative.

Although a causal relationship cannot be established from a single observation, the absence of an apparent local predisposing factor in our patient raises the possibility that external root resorption may represent an uncommon dental manifestation within the phenotypic spectrum of NS. Recent reports continue to emphasize the heterogeneous oral and maxillofacial manifestations of NS and the importance of careful dental assessment in affected patients [28]. Another clinically relevant aspect of NS is its association with an increased predisposition to certain malignancies, including juvenile myelomonocytic leukemia (JMML), neuroblastoma, rhabdomyosarcoma, and acute lymphoblastic leukemia [29,30]. Taken together, these findings highlight the importance of individualized long-term surveillance and multidisciplinary care in patients with NS, with consideration of the underlying molecular findings, overall clinical phenotype, and additional risk factors.

In this context, rhGH therapy represents an important therapeutic option for selected patients with NS and clinically significant growth impairment. Longitudinal studies have demonstrated improvements in growth velocity, height SDS, and adult height following rhGH treatment, particularly when therapy is initiated early in childhood [1,5,9,28]. Noordam et al. reported sustained acceleration of growth without significant deterioration in cardiac status during long-term treatment [31], while Osio et al. demonstrated improved final height outcomes in patients with NS treated with rhGH [10].

Nevertheless, given the multisystem nature of NS and its intrinsic association with an increased risk of certain malignancies, the long-term safety of rhGH therapy remains an important consideration. Current evidence does not indicate a substantial increase in malignancy risk directly attributable to rhGH treatment; however, appropriate cardiological assessment before treatment and continued clinical surveillance remain essential [29,30].

In our case series, rhGH therapy was initiated in selected patients (Patients 1, 5, 6) with clinically significant short stature following appropriate clinical and cardiological evaluation. However, the assessment of growth response and long-term treatment outcomes was beyond the scope of the present study, which was primarily intended to illustrate the phenotypic diversity of NS in pediatric patients. Further longitudinal follow-up will be required to evaluate individual growth responses, as well as the long-term effectiveness and safety of rhGH therapy in these patients.

Several limitations of this study should be acknowledged. First, the retrospective case-series design and small number of patients preclude generalization of the observed phenotypic patterns to the broader NS population. Second, the patients were selected to illustrate clinical heterogeneity and therefore do not represent an unselected cohort of children with NS. Endocrine, skeletal, and biochemical assessments were performed according to individual clinical indications and were consequently not uniform across all patients. In addition, detailed molecular analyses and genotype–phenotype correlations were not addressed, as the study was primarily focused on clinical and phenotypic characteristics. Similarly, although rhGH therapy was initiated in selected patients, evaluation of treatment response and outcomes was not an objective of the present case series. Nevertheless, the detailed clinical characterization of these patients illustrates diagnostically relevant phenotypic variability encountered in pediatric endocrine practice.

## 5. Conclusions

NS is characterized by remarkable phenotypic variability, ranging from the classical presentation with characteristic facial dysmorphism, congenital heart defects, and growth impairment to atypical or oligosymptomatic forms dominated by individual clinical manifestations. The cases presented in this series illustrate this broad spectrum, encompassing severe short stature, low bone mass, unusual dental abnormalities, relatively preserved linear growth, and phenotypes further modified by coexisting genetic conditions.

Importantly, the absence of characteristic dysmorphic or cardiovascular features, or even marked growth impairment, does not exclude NS. This variability is particularly relevant in pediatric endocrinology, where short stature, altered growth patterns, or other apparently isolated findings may be the predominant reason for clinical evaluation. Moreover, coexisting genetic conditions may substantially modify the expected phenotype and should be considered when clinical findings appear unusual or discordant.

These observations emphasize the importance of recognizing NS beyond its classical phenotype and support an individualized, multidisciplinary approach to patient evaluation and long-term follow-up. In selected children with persistent unexplained growth abnormalities, skeletal or dental findings, or other atypical manifestations that remain unexplained after conventional evaluation, NS should be considered in the differential diagnosis, with genetic testing used when clinically appropriate. Greater awareness of the full phenotypic spectrum of NS may facilitate earlier recognition, may help shorten otherwise prolonged diagnostic pathways, and enable more appropriately tailored multidisciplinary care.

## Figures and Tables

**Table 1:** Phenotypic spectrum and major clinical characteristics of patients with NS.

Patient	Sex/Age	Main Clinical Presentation	Growth Phenotype	Key Endocrine/Skeletal Findings	Associated Features/Conditions	Molecular Finding	Management
1	M/8 y	Classical NS phenotype with facial dysmorphism	Severe short stature	Normal stimulated GH secretion	Pulmonary valve stenosis, typical facial features	PTPN11	rhGH therapy
2	F/4 y	Classical NS phenotype	Relatively preserved growth	No significant endocrine abnormalities	Typical facial features, WPW syndrome	PTPN11	Clinical follow-up
3	M/14 y	Progressive external root resorption of permanent incisors	Normal stature	No significant endocrine abnormalities	Unusual dental phenotype	PTPN11, NBN	Multidisciplinary follow-up
4	M/8 y	NS phenotype modified by coexisting Klinefelter syndrome	Relatively preserved growth	No significant endocrine abnormalities	Facial dysmorphism, mitral valve insufficiency, Klinefelter syndrome	LZTR1; 47,XXY karyotype	Multidisciplinary follow-up
5	F/14 y	Predominantly severe growth impairment without classical NS dysmorphia	Severe short stature	Low IGF-1 with preserved GH secretion; markedly reduced BMD	Low bone mass	PTPN11	rhGH therapy
6	F/12 y	Predominantly growth impairment without classical NS dysmorphia	Short stature	Persistently low IGF-1 with preserved GH secretion	Intrafamilial phenotypic variability	PTPN11	rhGH therapy

**Table 2:** Auxological, pubertal and biochemical characteristics of the patients with NS.

Parameter	Patient 1	Patient 2	Patient 3	Patient 4	Patient 5	Patient 6
Height [cm]	105.60	96	156	135.90	145.40	139.60
Height percentile and height SDS	<3 hSDS = −4.49	3	10–25	25–50	<3 hSDS = −2.90	<3 hSDS = −2.10
Weight [kg]	16.60	14.80	48.00	29.70	28.70	30.30
BMI [kg/m^2^] SDS	14.89 SDS = −0.72	16.10 SDS = 0.34	19.72 SDS = 0.18	16.08 SDS = −0.06	13.58 SDS = −3.27	15.55 SDS = −1.10
Tanner stage	I	I	III	I	II	II
IGF-1 [ng/mL]	55.70 (RR: 40.1–255)	102.90 (RR: 53–216)	536.80 (RR: 120–501)	113.90 (RR: 85.7–343)	134 (RR: 151–485)	114.6 (RR: 140–468)
Peak GH stimulation test [ng/mL]	19.00	11.50	N/A	N/A	12.78	16.94
Serum calcium RR: 2.20–2.70 [mmol/L]	2.50	2.48	2.55	2.54	2.40	2.41
Serum phosphate RR: 1.09–2.00 [mmol/L]	1.36	1.76	1.74	1.98	1.61	1.66
Alkaline phosphatase [U/L]	N/A	145.00 (RR:160–381)	311.00 (RR: 130–534)	206.00 (RR: 160–381)	155.00 (RR: 62–288)	148.00 (RR: 144–475)
PTH RR: 15–65 [pg/mL]	N/A	22.30	27.40	18.40	33.60	38.50
25(OH) vitamin D [ng/mL]	24.81	26.10	32.50	22.90	25.60	18.90
DXA aBMD_for age_ Z-Scores	N/A	N/A	TBLH = −0.50 Spine = −0.80	TBLH = −2.30 Spine = −2.10	TBLH = −4.80 aBMD_HAZ_ Z-score TBLH = −3.38 Spine = −4.90 aBMD_HAZ_ Z-score Spine = −3.78	N/A

N/A—Not applicable.

## Data Availability

The datasets used and/or analyzed within the framework of this study are available from the corresponding author upon reasonable request. The data are not publicly available due to privacy and ethical restrictions related to patient confidentiality.
